# Effect of divalent and trivalent metal ions on artificial membrane permeation of fluoroquinolones^1^

**DOI:** 10.5599/admet.1427

**Published:** 2022-09-07

**Authors:** Nanami Nakatani, Kiyohiko Sugano

**Affiliations:** Molecular Pharmaceutics Lab., College of Pharmaceutical Sciences, Ritsumeikan University, 1-1-1, Noji-higashi, Kusatsu, Shiga 525-8577, Japan

**Keywords:** artificial membrane, permeability, phospholipid, fluoroquinolone, metal, cation

## Abstract

The purpose of the present study was to evaluate the predictability of PAMPA for the effect of metal ions on the bioavailability of fluoroquinolones (FQ). Eleven FQs and seven metal ions were employed in this study. The PAMPA membrane consisted of a 10 % soybean lecithin (SL) – decane solution. A drug solution in MES buffer with or without a metal ion (added as a chloride salt) was added to the donor compartment. In the absence of metal ions, FQ showed relatively high permeability (> 5 × 10^-6^ cm/sec) in SL-PAMPA despite their hydrophilic and zwitterionic properties. As the PAMPA permeability ratio with/without metal ions became smaller, the urinary excretion and AUC ratios tended to be smaller, suggesting that SL-PAMPA is a suitable in vitro model to evaluate the potential effect of metal ions on the bioavailability of FQ. However, the reduction in AUC and urinary excretion was overestimated for low solubility metal ion formulations (dried aluminum hydroxide gel and La_2_(CO_3_)_3_・8H_2_O). In such cases, the dissolution of the metal ion formulations and the permeation of FQs should be simultaneously evaluated.

## Introduction

Multivalent metal cations are present in many pharmaceutical and nutraceutical products, such as antacids, phosphate binders, and iron supplements. The concomitant administration of these multivalent metal salt formulations can reduce the bioavailability of various drugs, such as fluoroquinolones (FQ) [1,2], tetracyclines [3], and HIV-integrase inhibitors [4]. In an acidic environment of the stomach, a metal cation exists as a dissociated ion. However, it could form a complex with a drug molecule in a neutral pH environment of the small intestine. It is well known that multivalent metal cations reduce the intestinal membrane permeation of some FQs by chelate formation [2]. To avoid FQ - multivalent cation interactions in the gastrointestinal tract, it is recommended to separate the drug administration times [5]. However, this may decrease patient medication adherence. Currently, more than a dozen FQs and several multivalent metal salt formulations are prescribed in clinical practice. However, it is practically impossible to examine the effect of metal salt formulations on the bioavailability of FQs for all combinations by clinical studies.

The parallel artificial membrane permeation assay (PAMPA) has been widely used to assess the passive membrane permeation of drugs [6–9]. Recently, we reported that the membrane permeation of tetracyclines and the effect of metal ions can be evaluated using a phospholipid-based PAMPA [10,11]. However, it has been unknown whether PAMPA can predict the effect of metal salt formulations on the bioavailability of FQs.

**Figure 1.**
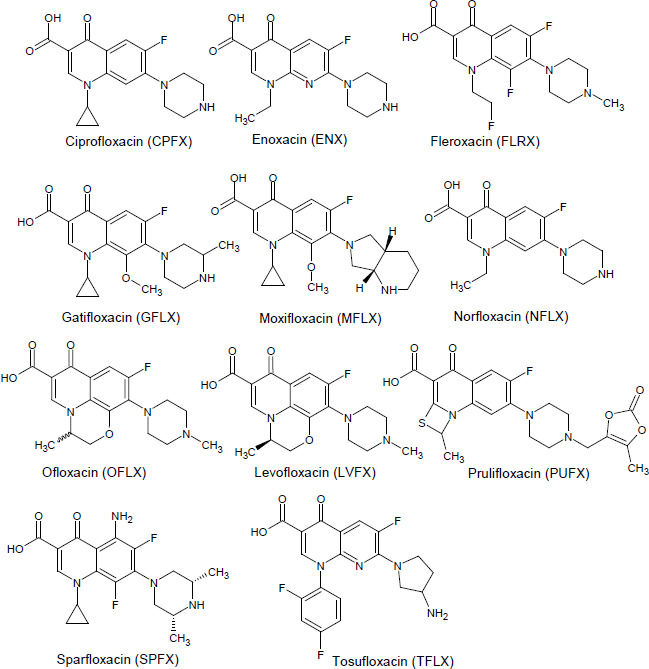
Chemical structures of fluoroquinolones used in this study

The purpose of the present study was to evaluate the predictability of PAMPA for the effect of metal ions on the bioavailability of FQs. Eleven FQs and seven metal ions are employed in this study (Figure 1, Table 1).

## Experimental

### Materials

Norfloxacin (NFLX), ofloxacin (OFLX), tosufloxacin tosylate monohydrate (TFLX), calcium dichloride, magnesium dichloride, iron(II) dichloride tetrahydrate, iron(III) chloride hexahydrate, aluminum(III) chloride hexahydrate, zinc chloride, lanthanum chloride heptahydrate, 1-octanol, sodium dihydrogen phosphate dihydrate, sodium chloride, sodium hydroxide, decane, 8 M NaOH, and 0.1 vol% trifluoroacetic acid-acetonitrile were purchased from Wako Pure Chemical Industries, Ltd (Osaka, Japan). Ciprofloxacin hydrochloride monohydrate (CPFX), levofloxacin (LVFX), enoxacin sesquihydrate (ENX), fleroxacin (FLRX), and prulifloxacin (PUFX) were purchased from TCI (Tokyo, Japan). Gatifloxacin (GFLX) and sparfloxacin (SPFX) were purchased from Funakoshi Co., Ltd (Tokyo, Japan). Moxifloxacin (MFLX) was purchased from Combi-Blocks Inc. (San Diego, USA). 2-Morpholinoethanesulfonic acid (MES) was purchased from Dojindo laboratories (Tokyo, Japan). 0.1 vol% trifluoroacetic acid-distilled water was purchased from Kanto chemical co., inc (Tokyo, Japan). Soybean lecithin was provided by Tsuji Oil Mills co., Ltd (Mie, Japan) (SLP-White, phosphatidylcholine (24-32 %), phosphatidylethanolamine (20-28 %), phosphatidylinositol (12-20 %), phosphatidic acid (8-15 %), and lysophosphatidylcholines (1-5 %) (based on the product information provided by the manufacturer)).

### Methods

#### PAMPA assay

The PAMPA sandwich consisted of a 96-well filter plate (hydrophobic PVDF, 0.45 μm) and a PAMPA acceptor plate (Merck Millipore, MA, USA). The acceptor plate was filled with 300 μL of a 50 mM MES buffer solution (pH 6.5). The filter bottom of the donor well was coated with 5 μL of a 10 % soybean lecithin (SL) – decane solution prepared in our laboratory [11]. The soybean lecithin–decane membrane (SL–PAMPA) was used because it most likely mimics the intestinal membrane [17].

A drug solution (0.5 mM in pH 6.5 50 mM MES buffer, 200 μL) with or without a metal ion was added to the donor compartment. The PAMPA sandwich was then incubated at 37 °C for 3 h without stirring. After incubation, 150 μL of both the donor and acceptor solutions were transferred to a UV plate. The concentrations of FQs were measured by UV absorption except for TFLX (Table 1). The concentration of TFLX was measured by HPLC (LC-20AD, Shimazu Corporation, Kyoto, Japan) (Column: ZORBAX Eclipse Plus C18 Narrow Bore RR 2.1×50 mm column with 3.5 μm particles (Agilent Technologies, CA); flow rate: 0.60 mL/min; mobile phase: 20 % 0.1 vol% trifluoroacetic acid-acetonitrile, 80 % 0.1 vol% trifluoroacetic acid-distilled water (isocratic elution); detection wavelength: 270 nm; column temperature: 40 °C ; injection volume: 10 μL). The PAMPA permeability (*P_e_*) was measured in triplicate. The *P_e_* value was calculated by the following equations [17].


(1)

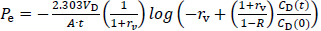




(2)

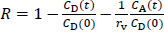




(3)

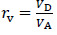



where *P_e_* is the effective permeation coefficient (cm/s), *A* is the filter surface area (0.266 cm^2^), *V_D_* and *V_A_* are the volumes (mL) of the donor and acceptor phases, respectively, *t* is the incubation time, *C_D_(t)* is the concentration of a drug in the donor phase at time *t*, *R* is the membrane retention factor, and *r_v_* is the volume ratio. These equations are generally used to calculate *P_e_* for PAMPA in the literature. The details for the derivation of these equations have been reported elsewhere [17]. In Equations 1 to 3, the mass balance of a drug in the donor fluid, the acceptor fluid, and the membrane are considered. The *R* values ranged from < 0.1 (CPFX, ENX, NFLX, OFLX, LVFX) to 0.36 (TFLX) in this study. Because it is difficult to collect samples multiple times from a 96-well plate, PAMPA *P_e_* is usually calculated from one-time point data. In typical PAMPA experiments, the steady state is achieved within 0 to 45 min [17]. Therefore, PAMPA *P_e_* is usually calculated from one-time point data at > 2 h [6–9].

#### Log *D* measurement of the fluoroquinolones

The octanol-buffer distribution coefficient (log *D*) was determined at pH 6.5 by a shake-flask method. The octanol and buffer phases were mutually pre-saturated before use. A buffer solution of a model drug (1.0 mM, 0.5 mL, 50 mM sodium – phosphate buffer) and octanol (2.5 mL) was added to a 15 mL tube. The sample was vigorously shaken for 90 min at 25 °C. Because log *D* is a physicochemical property of a drug, it was measured at a standard condition of 25 °C, rather than 37 °C. The concentration of FQs in the aqueous phase was determined as described above. Log *D* was measured in triplicate.

## Results

### Effect of physicochemical properties of fluoroquinolones on PAMPA permeability

**Figure 2.**
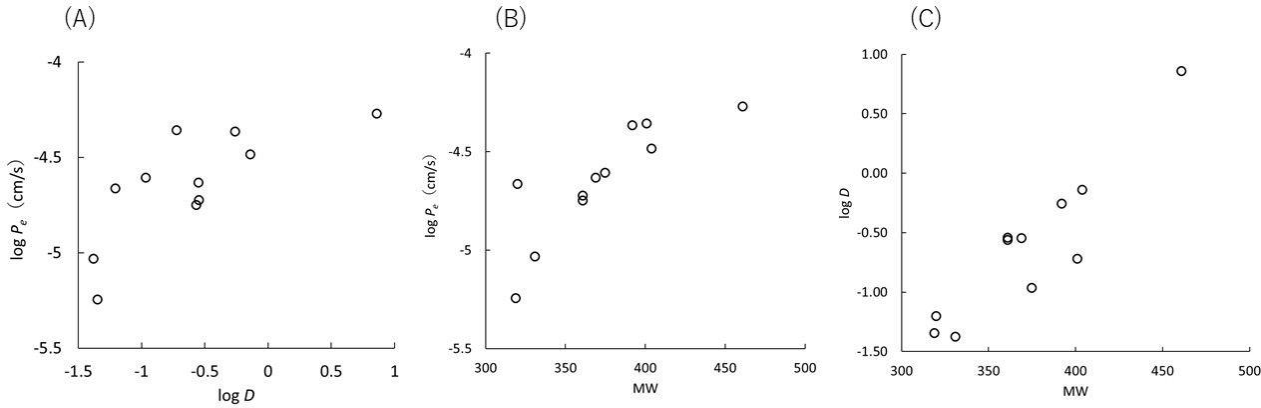
Correlation between PAMPA permeability, log *D*, and MW

All PAMPA permeability data are summarized in the supplemental material (Table S1). The effect of the physicochemical properties of FQs on PAMPA *P*_e_ was first investigated (Figure 2). The *P*_e_ value tended to increase as log *D* increased. Above log *D* > 0, the *P*_e_ value reached a plateau value of about 30 × 10^-6^ cm/s. There was no correlation between *P*_e_ and the p*K*_a_ values.

### In vitro – in vivo correlation of FQ – metal ion interaction

**Figure 3.**
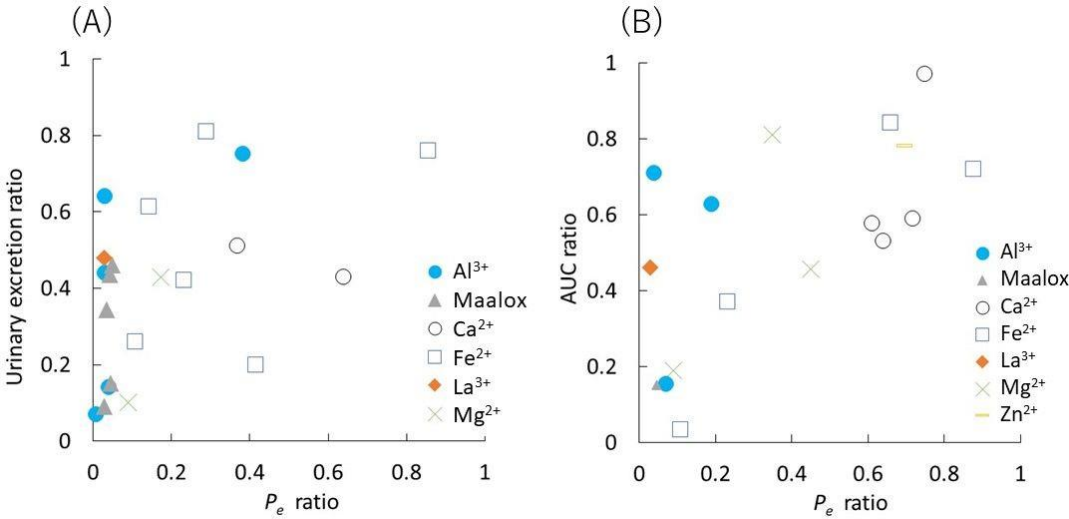
Correlation between PAMPA *P*_e_ ratio with/without metal ions and (A) urinary excretion ratio and (B) AUC ratio in humans (annotated by metal ions).

Since metal ions can interact with phosphate and citrate ions, MES buffer was used in this study. The concentration of metal ions was set to reflect the clinical dose and the gastrointestinal fluid volume. In the presence of metal ions, the *P*_e_ values were reduced in all FQs (supplemental material Table S1). Trivalent ions (Al^3+^, Fe^3+^, and La^3+^) tended to show a stronger inhibition effect than divalent ions. The correlations between the urinary excretion (Ur) and AUC ratios in humans and the PAMPA *P*_e_ ratio are shown in Figure 3 (categorized by metal ions) and also in Supplemental Material Figure S1 (categorized by FQ). Clinical and in vivo canine data are summarized in Supplemental Material Table S2 with the references. There is a general trend that as the *P*_e_ ratio (with/without metal ions) becomes smaller, the reduction in AUC and Ur becomes more significant. However, PAMPA tended to overpredict the reduction in AUC and Ur, especially for Al^3+^ and La^3+^ with high permeability FQs (*P*_e_ > 10^-5^ cm/s).

**Figure 4.**
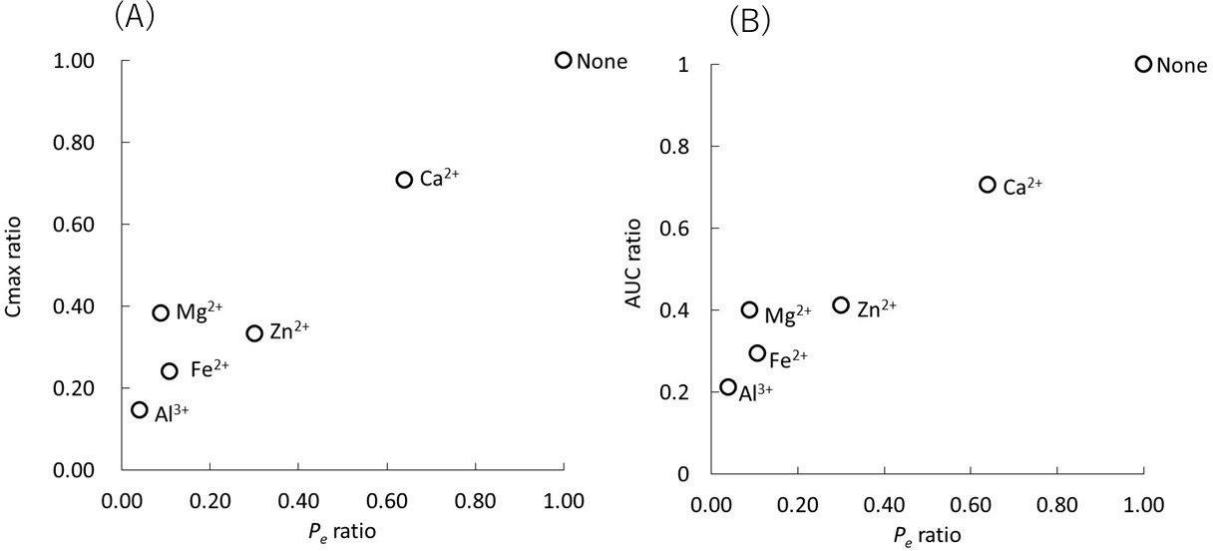
Correlation between the PAMPA *P*_e_ ratio and (A) *C*_max_ and (B) AUC ratios in dogs.

The interactions between norfloxacin (NFLX) and various metal ions have been investigated in dogs [18]. There is a good correlation between the PAMPA *P*_e_ ratio and the in vivo *C*_max_ and AUC ratios (Figure 4).

## Discussion

At pH 6.5, FQ molecules exist as a zwitterion (Table 1). According to the pH-partition hypothesis, zwitterionic drugs are often assumed to show poor membrane permeability [19]. However, we previously reported that zwitterionic tetracyclines can permeate the PAMPA membrane in the presence of phospholipids (soybean lecithin) (SL-PAMPA), despite their hydrophilic and zwitterionic properties [11]. In addition, SL-PAMPA has been used to investigate the effect of metal ions on the permeation of tetracyclines [10]. Therefore, SL-PAMPA was used in this study.

In the absence of metal ions, FQs showed relatively high permeability (> 5 × 10^-6^ cm/sec) in SL-PAMPA despite their hydrophilic and zwitterionic properties. The high *P*_e_ values of FQs in SL-PAMPA are in good agreement with that these FQs show good oral bioavailability. Even in the case of most hydrophilic FQs, NFLX (log *D* = - 1.35) and CPFX (log *D* = - 1.38), the bioavailability is moderate and high (> 38 % and 83 %, respectively) [20,21]. These results suggested that SL-PAMPA is suitable to assess the effect of metal ions on the intestinal membrane permeation of zwitterionic drugs. The *P*_e_ values of NFLX and CPFX are about 10 times higher than previously reported values in pION’s PAMPA, which uses a 20 % dodecane solution of a lecithin mixture [19,22]. As log *D* was increased, the *P*_e_ value was increased until reaching the plateau value (about 30 × 10^-6^ cm/s at log *D* > 0) (Figure 2A). This plateau value would be attributed to the unstirred water layer adjacent to the PAMPA membrane [23].

As shown in Figure 2B, PAMPA *P*_e_ was also positively correlated with MW. The observed correlation between log *P*_e_ and MW may be a mere coincidence due to the choice of FQs, but it may also be explained as follows. According to the solubility-diffusion theory [24,25], partition and diffusion processes determine the lipid membrane permeation of a drug. As MW increases, the diffusion coefficient decreases. On the other hand, the partition process depends on the lipophilicity of a drug. The lipophilicity of a drug is determined by the balance of drug-solvent interactions (e.g., hydrogen bonding) and the cavity effect, the latter of which is proportional to MW. Therefore, when drug-solvent interaction is similar (like among FQ series), log *D* increase with MW (Figure 2C). In most cases of drug membrane permeation, the influence of a partition process is greater than that of a diffusion process. It is well known that the membrane permeability of drugs correlates with their lipophilicity [9]. Therefore, even though an increase in MW decreases the diffusion process, *P*_e_ can show a positive correlation with MW. More detailed analysis is required to further investigate this point, such as using Abraham’s solute descriptors [26].

In this study, metal ions are added as chloride salts because they are soluble in aqueous media. The *P*_e_ ratio of NFLX was in good agreement with the *C*_max_ and AUC ratios observed in dogs (Figure 4) [18]. In that study, NFLX was administered as a 0.01 N HCl solution with the metal chloride salts. Therefore, the dissolution process of FQs and multivalent metal salt formulations were neglected. The effect of Mg^2+^ in dogs was slightly overestimated by PAMPA. The reason for this was not clear.

However, in clinical cases where these formulations are administered as solid dosage forms, PAMPA tended to overpredict the reduction of AUC and *C*_max_, especially for Al^3+^ and La^3+^ formulations (Figure 3). In clinical formulations, Al^3+^ is contained as dried aluminum hydroxide gel and La^3+^ is contained as La_2_(CO_3_)_3_・8H_2_O. Ca^2+^, Mg^2+^, Fe^2+^, and Zn^2+^ are also formulated as the other solid forms (CaCO_3_, MgO, etc.). Some of these solid forms would dissolve slowly in the intestinal fluid so FQs can be absorbed before metal ions are released from the formulations, especially for high permeability FQs. For example, the dissolution of La_2_(CO_3_)_3_・8H_2_O is about 50 % at 15 min in the gastric environment of pH 1.2 and there is little dissolution in the intestinal environment of pH 6.8 [27]. For more quantitative prediction, the dissolution of a metal ion formulation and the permeation of an FQ should be simultaneously evaluated.

The detailed analysis of equilibria between FQs and metal ions is out of the scope of this study, as it has been reported for some of the FQs [28]. Previously, Wallis et al. reported that the formation constant of the NFLX – metal ion complex correlates with in vivo bioavailability in dogs [18]. The chelate formation constant (log unit) and the percentage of NFLX complexed at pH 6.5 are reported to be 2.2 (8.5 %), 3.0 (35 %), 3.8 (81 %), 4.0 (78 %), and 7.0 (99 %) for Ca^2+^, Mg^2+^, Zn^2+^, Fe^2+^, and Al^3+^, respectively. In the present study, the trivalent metal ions (Al^3+^, Fe^3+^, La^3+^) reduced *P*_e_ more than the divalent metal ions. Among the divalent metal ions, no general trend was observed, except that Mg^2+^ reduced *P*_e_ more than Ca^2+^ in all FQs. This trend was not observed in tetracyclines [10].

## Conclusion

In conclusion, the *P*_e_ values of FQs are relatively high in SL-PAMPA. As the *P*_e_ ratio (with/without metal ions) becomes smaller, the reduction in AUC and Ur tended to be more significant. However, the reduction in AUC and Ur is overestimated for low solubility metal ion formulations (especially Al^3+^ and La^3+^ formulations). In such cases, the dissolution of a metal ion formulation and the permeation of an FQ should be simultaneously evaluated for more quantitative prediction by an in vitro experiment. SL-PAMPA is suitable to evaluate the potential effect of metal salt formulations on the bioavailability of FQs.







## Figures and Tables

**Table 1.** Physicochemical properties of fluoroquinolones used in this study

Drug	MW	p*K_a_*	log *D* (pH 6.5)^a^	UV (nm)	p*K_a_* Ref.
Ciprofloxacin (CPFX)	331	6.1, 8.7	-1.38 ± 0.15	335	[12]
Enoxacin (ENX)	320	6.3, 8.7	-1.20 ± 0.39	330	[12]
Fleroxacin (FLRX)	369	5.5, 8.1	-0.55 ± 0.10	283	[12]
Gatifloxacin (GFLX)	375	5.7, 8.7	-0.97 ± 0.04	330	[13]
Levofloxacin (LVFX)	361	6.1, 8.2	-0.56 ± 0.11	335	[14]
Moxifloxacin (MFLX)	401	6.4, 9.5	-0.72 ± 0.38	350	[12]
Norfloxacin (NFLX)	319	6.3, 8.4	-1.35 ± 0.17	280	[12]
Ofloxacin (OFLX)	361	6.1, 8.2	-0.55 ± 0.12	330	[12]
Prulifloxacin (PUFX)	461	5.6, 6.3	0.86 ± 0.02	283	[15]
Sparfloxacin (SPFX)	392	6.3, 8.8	-0.26 ± 0.03	300	[12]
Tosufloxacin (TFLX)	404	5.8, 8.7	-0.14 ± 0.10	270	[16]

^a^ Measured in this study. Mean ± SD (N = 3)
