# Supplementary material for: Effect of divalent and trivalent metal ions on artificial membrane permeation of fluoroquinolones
Source: ADMET DMPK. 2022 Sep 7;10(4):289–97. doi: 10.5599/admet.1427 (PMC9793463; doi:10.5599/admet.1427)
Supplement: Supplementary file 1 [file ADMET-10-1427-S1.pdf]

Supplementary table

Effect of divalent and trivalent metal ions on artificial membrane permeation of fluoroquinolones

Nanami Nakatani, Kiyohiko Sugano*, https://doi.org/10.5599/admet.1427

Table S1 PAMPA permeability of fluoroquinolones in the absence or presence of metal ions

		P_e (10^{-6} cm/s)<br>P_e ratio [a]<br>Metal concentration (mM)[b]							
Drug	None	Ca^{2+}	Mg^{2+}	Fe^{2+}	Fe^{3+}	Al^{3+}	Zn^{2+}	La^{3+}	Mg^{2+} + Al^{3+}[d]
NFLX	5.7 ± 0.9	3.9 ± 0.4	1.5 ± 0.1	0.6 ± 0.1	0.4 ± 0.1	0.3 ± 0.0	0.6 ± 0.1	0.6 ± 0.1	0.2 ± 0.1
	-[c]	0.64	0.26	0.11	0.07	0.05	0.11	0.10	0.03
	-	5.0	5.0	5.0	5.0	5.0	5.0	5.0	10, 10 [b]
			0.5 ± 0.1				1.7 ± 0.1		
			0.09				0.30		
			10				0.5		
CPFX	9.3 ± 0.8	5.4 ± 0.4	3.7 ± 0.2	2.2 ± 0.4	1.5 ± 0.1	0.7 ± 0.1	1.2 ± 0.3	0.3 ± 0.1	0.4 ± 0.1
	-	0.58	0.40	0.23	0.16	0.07	0.13	0.03	0.04
	-	5.0	5.0	5.0	5.0	5.0	5.0	5.0	5, 5
		6.7 ± 0.3		2.3 ± 0.4			6.5 ± 0.5		
		0.72		0.25			0.70		
		1.7 [b]		1.0 [b]			0.033 [b]		
LVFX	17.8 ± 0.2	13.3 ± 0.6	8.7 ± 0.4	3.4 ± 0.5	2.7 ± 0.4	2.0 ± 0.1	3.9 ± 0.3	0.5 ± 0.1	1.2 ± 0.1
	-	0.75	0.49	0.19	0.15	0.11	0.22	0.03	0.07
	-	5.0	5.0	5.0	5.0	5.0	5.0	5.0	5, 5
OFLX	18.9 ± 0.2	15.6 ± 0.9	9.3 ± 0.2	3.6 ± 0.2	2.1 ± 0.3	2.4 ± 0.1	4.3 ± 0.4	0.2 ± 0.0	1.1 ± 0.1
	-	0.83	0.49	0.19	0.11	0.13	0.23	0.01	0.06
	-	5.0	5.0	5.0	5.0	5.0	5.0	5.0	5, 5
				16.3 ± 0.2		0.6 ± 0.1			
				0.86		0.03			
				0.30 [b]		12.5 [b]			
ENX	21.7 ± 0.8	15.6 ± 0.7	8.1 ± 0.6	6.1 ± 1.0	3.0 ± 0.2	1.2 ± 0.2	2.6 ± 0.1	0.02 ± 0.0	0.9 ± 0.1
	-	0.72	0.37	0.28	0.14	0.05	0.12	0.001	0.04
	-	5.0	5.0	5.0	5.0	5.0	5.0	5.0	10, 10 [b]
MFLX	43.9 ± 2.7	30.4 ± 1.2	15.8 ± 0.6	6.3 ± 0.5	4.8 ± 0.9	1.4 ± 0.1	4.7 ± 0.2	0.4 ± 0.0	2.2 ± 0.2
	-	0.70	0.36	0.14	0.11	0.03	0.11	0.01	0.05
	-	5.0	5.0	5.0	5.0	5.0	5.0	5.0	10, 10 [b]
TFLX	32.7 ± 2.6	20.3 ± 7.8	14.7 ± 6.3	21.6 ± 0.9	4.5 ± 0.5	6.2 ± 0.6	8.0 ± 0.8	1.0 ± 0.0	3.9 ± 0.3
	-	0.61	0.45	0.66	0.14	0.19	0.25	0.03	0.12
	-	5.0	5.0	5.0	5.0	5.0	5.0	5.0	10, 10 [b]

a The Pe ratio with/ without metal ions; b Metal ion concentration was set to 5 mM or the clinical condition (Dose/ 250 mL water) when clinical data are available; c Not applicable.; d Maalox®

Table S1 Continued

Drug	None	Ca^{2+}	Mg^{2+}	Fe^{2+}	Fe^{3+}	Al^{3+}	Zn^{2+}	La^{3+}	Mg^{2+} + Al^{3+}[d]
					P_e (10^{-6} cm/s)<br>P_e ratio [a]<br>Metal concentration (mM)[b]				
FLRX	23.3 ± 5.1	13.8 ± 0.9	8.3 ± 0.3	8.5 ± 1.6	3.3 ± 0.3	1.9 ± 0.1	2.7 ± 0.1	0.2 ± 0.0	1.9 ± 0.1
	-	0.62	0.37	0.36	0.14	0.09	0.12	0.01	0.08
	-	5.0	5.0	5.0	5.0	5.0	5.0	5.0	5, 5
						8.9 ± 0.4			
						0.38			
						0.25 [b]			
PUFX	53.7 ± 13.6	33.1 ± 2.1	18.3 ± 0.2	2.8 ± 1.0	8.7 ± 1.9	1.6 ± 0.5	8.2 ± 3.0	5.4 ± 0.4	2.1 ± 0.9
	-	0.63	0.35	0.05	0.17	0.03	0.16	0.10	0.04
	-	5.0	5.0	5.0	5.0	5.0	5.0	5.0	5, 5
		19.9 ± 3.9	9.1 ± 0.1	22.6 ± 4.4		0.5 ± 0.2			
		0.37	0.17	0.42		0.01			
		10 [b]	10 [b]	0.25 [b]		10 [b]			
GFLX	24.7 ± 2.2	17.2 ± 0.2	10.5 ± 0.3	7.0 ± 0.4	6.1 ± 0.5	2.2 ± 0.1	3.1 ± 0.2	2.3 ± 0.1	1.0 ± 0.1
	-	0.70	0.43	0.29	0.25	0.09	0.13	0.09	0.04
	-	5.0	5.0	5.0	5.0	5.0	5.0	5.0	5.75, 5.25 [b]
				18.0 ± 1.0					
				0.73					
				0.4 [b]					
SPFX	43.1 ± 0.7	28.6 ± 1.2	14.9 ± 0.4	10.0 ± 0.8	6.8 ± 0.4	1.6 ± 0.2	3.8 ± 0.2	1.4 ± 0.2	2.2 ± 0.1
	-	0.66	0.35	0.23	0.16	0.04	0.09	0.03	0.05
	-	5.0	5.0	5.0	5.0	5.0	5.0	5.0	4.25, 5.25 [b]
				37.9 ± 3.9					
				0.88					
				0.2 [b]					
