# Supplementary material for: Effect of divalent and trivalent metal ions on artificial membrane permeation of fluoroquinolones
Source: ADMET DMPK. 2022 Sep 7;10(4):289–97. doi: 10.5599/admet.1427 (PMC9793463; doi:10.5599/admet.1427)
Supplement: Supplementary file 3 [file ADMET-10-1427-S3.pdf]

Supplementary Figure

Effect of divalent and trivalent metal ions on artificial membrane permeation of fluoroquinolones

Nanami Nakatani, Kiyohiko Sugano*, https://doi.org/10.5599/admet.1427

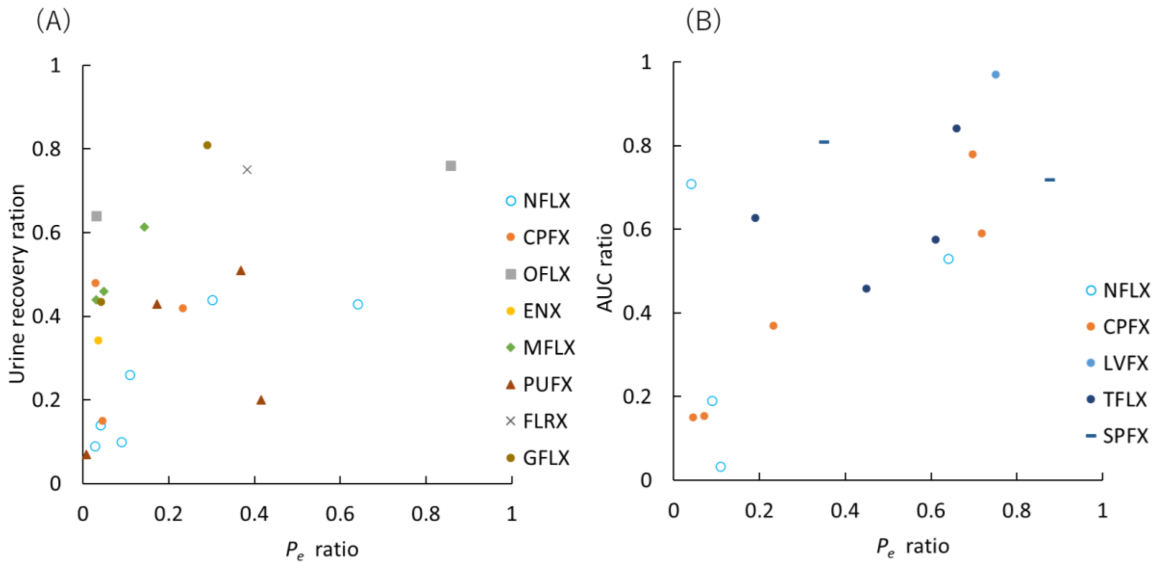

Figure S1 Correlation between PAMPA P_e ratio with/without metal ions and (A) urinary excretion ratio and (B) AUC ratio in humans (annotated by FQs).
